# Effectiveness of exercise based on wearable electronic devices on lower limb strength and balance in older adults: a systematic review and meta-analysis

**DOI:** 10.3389/fpubh.2026.1778082

**Published:** 2026-02-25

**Authors:** Yaguang Li, Qingze Li, Xiaoxu Chen

**Affiliations:** 1 Physical Education College, Jiamusi University, Jiamusi, China; 2 College of Sports and Health, Shenyang Sport University, Shenyang, China; 3 Faculty of Chinese Medicine Science Guangxi University of Chinese Medicine, Nanning, China

**Keywords:** exercise, health, older adults, physical activity, wearable electronic devices

## Abstract

**Objective:**

This systematic review and meta-analysis aimed to evaluate the effectiveness of wearable electronic device–based exercise interventions on lower limb strength and balance in older adults.

**Methods:**

A systematic search was conducted following PRISMA guidelines to evaluate the impact of exercise based on wearable electronic devices on health behaviors (such as muscle strength, balance, endurance, mental health, and cognitive function) in older adults. This meta-analysis included 13 two-arm, between-group studies and 4 single-arm, within-group studies, involving a total of 611 participants. The inclusion of single-arm studies in the meta-analysis was based on the limited availability of two-arm studies and to maximize the available evidence for understanding the broader effects of the intervention.

**Results:**

The results show that in the meta-analysis of two-arm controlled studies, exercise based on wearable electronic devices significantly improved lower limb strength (SMD = −0.60; 95% CI [−1.15, −0.05]; *p* < 0.001; *I*^2^ = 81%) and balance (SMD = −0.43; 95% CI [−0.81, −0.06]; *I*^2^ = 17%) in older adults. However, the high heterogeneity for lower limb strength (*I*^2^ = 81%) should be interpreted cautiously, as it suggests substantial variability across studies. Subgroup analysis found that interventions with a frequency of once per week, a session duration of 10–45 min, and a total intervention duration of 8–12 weeks showed the best improvement in lower limb strength. Additionally, in the meta-analysis of single-arm studies, exercise based on wearable electronic devices significantly improved lower limb strength (SMD = 0.51; 95% CI [0.05, 0.97]; *I*^2^ = 23.5%). However, no significant effects were found on endurance, upper limb strength, mental health, cognitive function, and waist-to-hip ratio.

**Conclusion:**

The significant improvements in lower limb strength and balance, as key factors in older adults’ health, may have a positive impact on physical activity function, but falls were not directly meta-analyzed in this study. The findings support the potential of wearable electronic device-based exercise to improve specific aspects of health in older adults, but further studies are needed to confirm its broader impact on fall prevention and other health outcomes.

**Systematic review registration:**

PROSPERO, CRD420251273186.

## 1 Introduction

With the ongoing global aging process, the health and well-being of the older adults have become core issues in public health (1–4). As people age, they face complex health challenges, including decreased muscle strength (5), reduced balance (6), cognitive dysfunction (7), and an increased risk of various metabolic diseases (8). These declines not only reduce their independence and social participation but also lead to psychological problems, such as depression and loneliness, which severely impact their quality of life (9). The growing demand for older adults healthcare also places a significant socio-economic burden on families, communities, and healthcare systems (10, 11). Therefore, it is crucial to explore and promote accessible and sustainable health interventions to improve the overall health of older adults and delay functional decline.

Wearable electronic devices (e.g., smartwatches, activity trackers, and biosensor patches) offer a practical platform for implementing and monitoring exercise interventions in daily life. By enabling continuous, non-invasive measurement of activity and physiological signals (such as step counts, intensity distribution, heart rate, and sleep indicators) (12–15), wearables can translate general exercise recommendations into quantifiable goals and deliver timely feedback (16–18). These features may improve adherence through self-monitoring, goal setting, and motivational prompts, while also supporting remote supervision and safety monitoring—capabilities that are particularly relevant for older adults who may face mobility limitations or chronic conditions (15, 19, 20). As a result, wearable-supported exercise programs have become increasingly common in both community and clinical settings.

Exercise is a well-established non-pharmacological intervention that can improve physical performance, cardiometabolic health, and aspects of mental well-being in older populations (21–26). However, evidence specific to wearable-based exercise interventions remains difficult to synthesize for three reasons (17, 20). First, intervention designs vary substantially across studies (device type, feedback modality, behavior-change components, and intervention intensity/duration), which likely contributes to heterogeneous effects (17, 20, 27). Second, the evidence base includes both randomized controlled trials (RCTs) with a comparator (two-arm studies) and single-arm pre–post studies that are frequently used in feasibility, implementation, or early-phase research, excluding either design risks discarding a meaningful portion of the literature (28). Third, prior reviews have often focused on a limited set of outcomes, while wearable-supported exercise may plausibly influence multiple domains relevant to healthy aging (20, 29).

To address these gaps, we conducted a systematic review and meta-analysis to quantify the effects of wearable-supported exercise interventions on multidimensional health outcomes in older adults. We prespecified outcome domains that reflect established aging pathways and clinical relevance: (a) physical function and performance (e.g., strength, balance, and endurance), because they are central determinants of independence; (b) cardiometabolic and body composition indicators (including waist-to-hip ratio as a marker of central adiposity linked to cardiometabolic risk), because wearables commonly target activity behaviors that influence metabolic health; and (c) cognitive and mental health outcomes, given evidence that physical activity may support neurocognitive function and psychological well-being in later life. Methodologically, we integrated evidence from both two-arm trials and single-arm pre–post studies to provide a more complete estimate of effectiveness across efficacy- and implementation-oriented research, and we explored sources of heterogeneity related to intervention and device characteristics. Collectively, this work aims to clarify whether and in which domains wearable-supported exercise benefits older adults, and to inform future intervention design and research priorities.

## 2 Materials and methods

This review was performed in accordance with the Preferred Reporting Items for Systematic Reviews and Meta-Analyses (PRISMA) (30) guidelines and preregistered in the PROSPERO database (ID: CRD420251273186).

### 2.1 Search strategy

We conducted a search in three English databases (PubMed, MEDLINE, Web of Science), two Chinese databases (CNKI and WANFANG DATA), and one gray literature database (ProQuest) from the inception of the databases until December 25, 2025. A Medical Subject Heading (MeSH) search was performed to establish all relevant literature on physical activity using wearable electronic devices. Specifically, the database searches were performed using the following keywords and truncations in conjunction with the following search criteria: (Wearable Electronic Devices OR Wearable Electronic Device OR Wearable Devices OR Wearable Device OR Wearable Technology OR Wearable Technologies OR Electronic Skin OR Wearable Computer OR Wearable Computers) AND Exercise[MeSH] AND Aged[MeSH] AND English[lang]. Additionally, the reference lists of relevant meta-analyses and articles were also screened. Two reviewers independently assessed the identified publications for eligibility, with no language restrictions. Any disagreements were resolved through consensus. For specific retrieval strategies, refer to the Supplementary material.

### 2.2 Study selection

Studies were eligible if they met the following criteria: (1) Study design: (a) parallel-group randomized controlled trials or quasi-experimental studies with a comparator; and/or (b) single-arm pre–post studies included only for design-stratified, exploratory synthesis to summarize within-group changes where controlled evidence was limited. Importantly, two-arm and single-arm studies were not pooled together in the same meta-analysis. (2) Participants: older adults (≥60 years) with any health condition. (3) Intervention: any exercise/physical activity intervention delivered with the assistance of wearable electronic devices. (4) Comparator (for two-arm studies): usual care, normal daily activities, or exercise without wearable device assistance. (5) Outcomes: at least one quantitative measure relevant to older adult health, including lower-limb strength, balance, waist-to-hip ratio, cognitive function, endurance, or mental health.

### 2.3 Data extraction and effect-size preparation

Two reviewers independently extracted study characteristics and outcome data (means, standard deviations, and sample sizes). For two-arm studies, we extracted post-intervention values (or change scores when only change data were available) for intervention and comparator groups. For single-arm studies, we extracted pre- and post-intervention values to compute within-group changes. When necessary, authors were contacted for missing information; if essential data could not be obtained, the study was excluded from quantitative synthesis.

### 2.4 Methodological quality of included studies

The quality of the included studies was assessed using the Cochrane Risk of Bias tool, with particular attention to seven domains: random sequence generation, allocation concealment, blinding of participants and personnel, blinding of outcome assessment, completeness of outcome data, selective reporting, and other potential sources of bias. Each domain was rated as having a low risk, high risk, or unclear risk of bias according to the standards outlined in the Cochrane Handbook for Systematic Reviews (31), and any disagreements were resolved through consensus.

### 2.5 Statistical analysis

Meta-analyses were conducted using Review Manager 5.4 and StataMP 15. Continuous outcomes were synthesized using either mean difference (MD) when studies reported outcomes on the same scale and unit, or standardized mean difference (SMD) when different instruments/scales were used. Therefore, WMD was not used. Because internal validity and comparability differ across designs, we performed design-stratified meta-analyses: Two-arm controlled synthesis (primary analysis): effect sizes were computed as the between-group difference at post-intervention (intervention vs. comparator). When only pre–post change data were available, we used change-score SMD/MD as recommended in the Cochrane Handbook. Single-arm pre–post synthesis (exploratory analysis): effect sizes were computed as within-group pre–post changes. These results were interpreted as supportive/implementation-oriented evidence and were not combined with the two-arm estimates.

A random-effects model was used as the primary approach due to expected clinical and methodological variability (participants’ health status, intervention dose, device type, feedback/behavior-change components, and outcome measurement). Effect estimates are presented with 95% confidence intervals (CIs), and statistical significance was assessed using *Z*-tests (two-sided *α* = 0.05).

To ensure consistent interpretation across outcomes, we aligned effect directions *a priori*. Negative SMD/MD values were defined to indicate improvement for outcomes where a lower value reflects better status. Conversely, for outcomes where higher values represent improvement, data were entered so that improved performance corresponded to the same direction across studies.

To avoid unit-of-analysis errors when a study contributed multiple measures within the same outcome domain (e.g., multiple balance tests) or multiple time points, we applied the following rules: One effect size per study per outcome domain per synthesis. When multiple instruments assessed the same domain at the same time point, we prioritized the most prespecified/clinically common measure (e.g., for balance: Berg Balance Scale when available), or otherwise selected the measure identified as the primary outcome by study authors. If multiple eligible time points were reported, we used the immediate post-intervention assessment as the primary time point. Longer follow-up data were summarized narratively or analyzed in sensitivity analyses when sufficient studies were available. When two distinct subgroups within a single study were eligible, we combined groups using Cochrane-recommended formulas to avoid double-counting the comparator.

Statistical heterogeneity was assessed using the *I*^2^ statistic. We interpreted I^2^ values using conventional thresholds (25% low, 50% moderate, 75% high). When heterogeneity was very high (*I*^2^ > 75%), we implemented a prespecified interpretation plan: verify data extraction, effect direction harmonization, and outcome-domain classification; conduct influence (leave-one-out) analyses to identify outliers; perform subgroup analyses when ≥2 studies per subgroup were available; if substantial heterogeneity persisted and clinical/methodological diversity was judged to be too large for meaningful pooling, we refrained from meta-analysis for that outcome and provided a structured narrative synthesis instead. Publication bias was assessed when ≥8 studies were available for an outcome using funnel plots and Egger’s test (Stata).

## 3 Results

### 3.1 Study identification and selection

**Figure 1:**
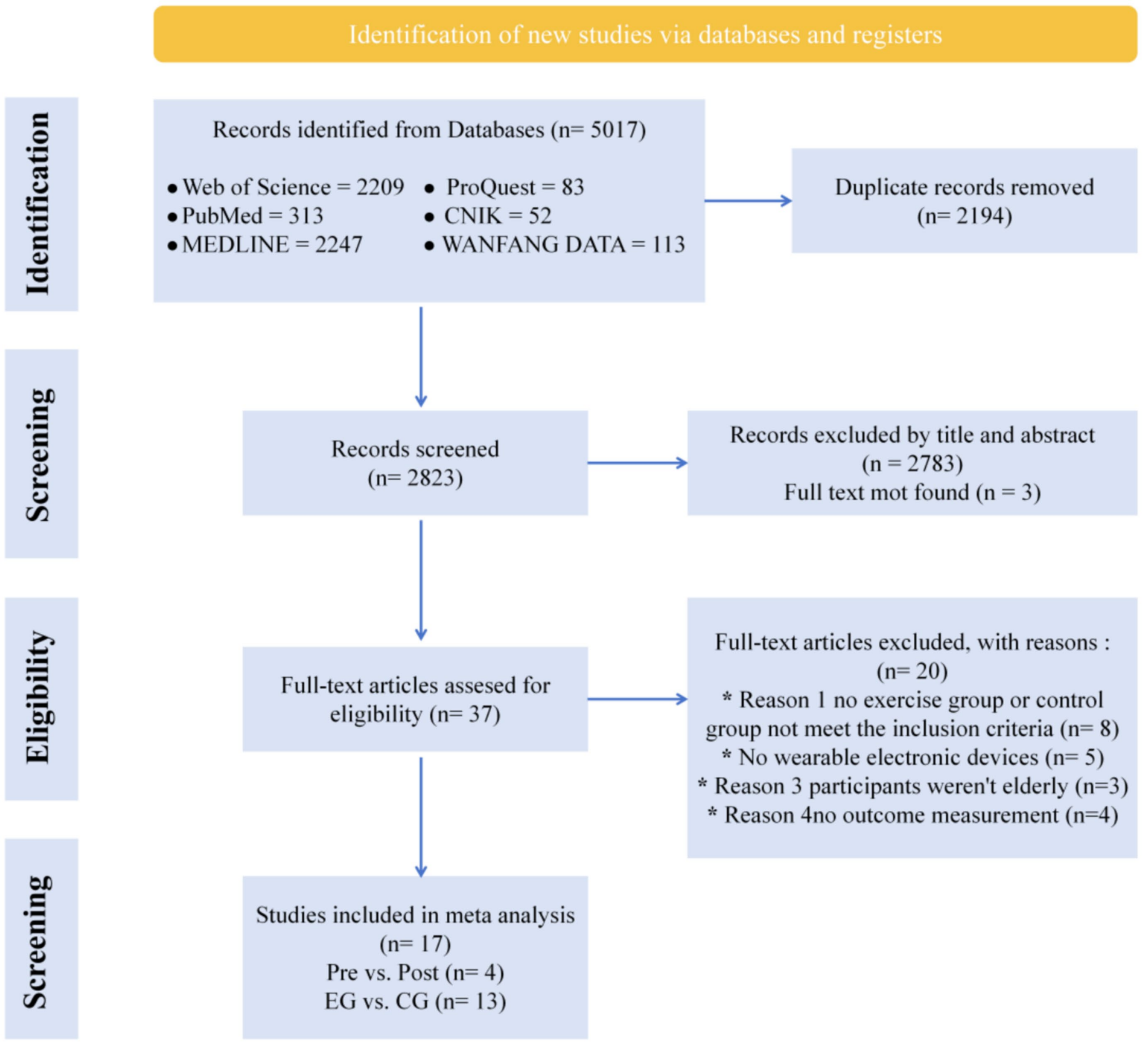
Flow chart diagram identifying the screening process and the studies included in the present review.

The initial database search yielded 5,017 publications. Subsequent screening resulted in 17 papers eligible to be included in the meta-analysis (Figure 1).

### 3.2 Study characteristics

**Figure 2:**
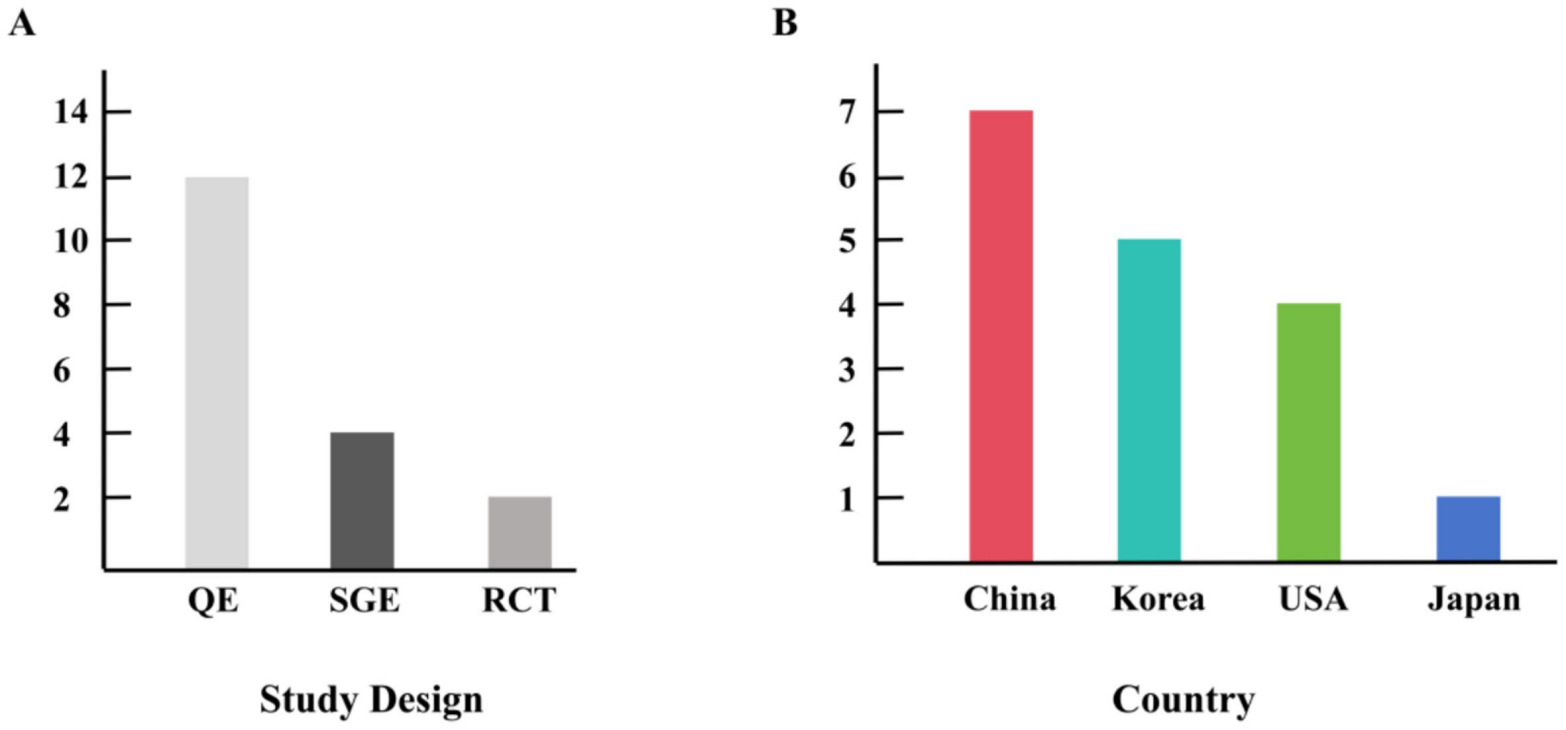
Research countries and study design information. QE, Quasi-experimental; SGE, single-group experiment; RCT, randomized controlled trial. **(A)** Study design included in the study, **(B)** Countries included in the study.

This study included 611 participants (age range: ≥60 years) from the included studies. The studies consist of 13 group comparison studies and 4 single-group analyses, which were mainly conducted in China, USA, Korea, and Japan (Figure 2). Detailed descriptions of the study participants are provided in Tables 1–3.

Table 1 provides an overview of the participant characteristics in the two-arm controlled trials included in this meta-analysis. The included studies involved healthy and frail populations, with participants aged 60 years and older. The sample size of the experimental group (EG) ranged from 9 to 40 participants, while the control group (CG) had a sample size ranging from 8 to 40 participants. The studies were conducted in multiple countries, including China, South Korea, the United States, and Japan, with designs including randomized controlled trials (RCTs) and quasi-experimental designs (QEs). The participants’ health status ranged from healthy and pre-frail to frail, and the outcomes assessed various aspects of physical health, including muscle strength, balance, endurance, and cognitive function. The diversity in sample characteristics, outcome measures, and measurement tools across these studies provides a broad basis for evaluating the impact of wearable device-based exercise interventions on the health outcomes of older adults.

**Table 1:** Characteristic of participants in the dual-arm controlled trial.

Study identification and basic characteristics			Characteristics of the study subjects			Output information		
Study	Country	Study design	Sample size (n); age (years)	Sex	Baseline health status	Outcome indicators	Measuring tools	Outcomes presented in the study
Wu and Manga (44)	China	QE	EG: 40; ≥65 CG: 40; ≥65	Male; Female	Healthy	Body composition; Muscle strength; Sarcopenia	Portable InBody 370; SFT; 5xSTS; MWT; ASMI based on BIA	Exhibited significant improvements in body composition and muscle strength.
Jung et al. (2025) (45)	Korea	RCT	EG: 18; ≥65 CG: 18; ≥65	Male; Female	Healthy	Cognitive functions; Dynamic balance; Muscle strength; Endurance	MoCA; TUG; ACT; GS; 5xSTS; MWT	Wearable ICMT improves cognitive-physical function and quality of life in older adults.
Shin et al. (2024) (46)	Korea	RCT	EG: 10; ≥65 CG: 12; ≥65	Male; Female	Healthy	Muscle power	SLS	Increased lower limb strength reduces the risk of falls among the older adults.
Roberts et al. (2019) (47)	USA	RCT	EG: 20; ≥60 CG: 22; ≥60	Male; Female	Healthy	Muscle power	4-m MWT; Hand grip Strength test	Wearable devices promote exercise and lower cardiovascular risk in older adults populations.
Lai et al. (2019) (48)	China	RCT	EG: 30; ≥60 CG: 30; ≥60	Male; Female	Pre-frailty stage	Muscle strength; physical fitness; energy metabolism	MRAK–10; SFT; wGT3X-BT	Wearable devices monitor health, resistance exercise counters frailty.
Schwenk et al. (2014) (49)	USA	RCT	EG: 15; ≥65 CG: 15; ≥65	Male; Female	Healthy	Body composition; Mental health; Motor performance	MMSE	Older adults people at risk of falls can benefit from balance training programs.
Liu et al. (2021) (50)	China	RCT	EG: 22; ≥65 CG: 18; ≥65	Male; Female	Frail	physical endurance	30STS; TUG; MWT	Wearable activity trackers with behavioral change techniques boost physical activity in younger adults.
Kannan et al. (2024) (51)	USA	RCT	EG: 13; ≥60 CG: 14; ≥60	Male; Female	Frail	Balance; Muscle strength; Endurance	FSST; 30STS; 2-min step in-place test	Remote exercise programs based on games can improve the physical functions of frail older adults people
Lee et al. (2025) (52)	China	RCT	EG: 16; ≥60 CG: 18; ≥60	Male; Female	Frail	Mental health	SWEMWBS	Outdoor exercise facility-based mobile health interventions benefit frail older adults
Yi et al. (2024) (53)	Korea	RCT	EG: 30; ≥60 CG: 30; ≥60	Male; Female	Healthy	Balance; Muscle strength;	TUG; MWT; 30STS	Smart healthcare, low-tech self-care exercise programs effectively promote community health and prevent falls in older adults.
Kwan et al. (2020) (54)	China	RCT	EG: 16; ≥60 CG: 17; ≥60	Male; Female	Frail	Balance; Muscle strength; Endurance	TUG; MWT; 30STS	Mobile health brisk walking intervention can help recognize the physical condition of vulnerable older adults people
Osuka et al. (2022) (55)	Japan	RCT	EG: 29; ≥65 CG: 29; ≥65	Male; Female	Frail	physical performance; mental health;	SPPB; MCS	The family Radio-Taiso exercise program improves the physical and mental health of frail older adults people
Kwan et al. (2021) (56)	China	RCT	EG: 9; ≥60 CG: 8; ≥60	Male; Female	Frail	Cognitive Function; Muscle strength	MoCA; TUG	VR motor-cognitive training enhances cognitive function in older adults with cognitive frailty

QE, Quasi-experimental; EG, Experimental Group; CG, Control group; SFT, Senior Fitness Test; RCT, randomized clinical trial; MoCA, Montreal cognitive assessment; TUG: Including: four-square step test (FSST), functional reach test (FRT), and single leg stance (SLS); ACT, arm curl test; GS, grip strength; STS, sit to stand test; MWT, minute walk test; MRAK-10, Digital muscle strength measuring instrument; SFT, senior fitness test; SWEMWBS, Short Warwick-Edinburgh Mental Well-Being Scale; SPPB, Short physical performance battery; MCS, mental component summary; MMSE, cognitive status.

Table 2 summarizes the intervention protocols in the two-arm controlled trials included in the meta-analysis. The table provides detailed information about the intervention methods, including the types of electronic devices used, the nature of physical or cognitive training, the frequency and duration of the interventions, and the intensity levels. Most studies involved structured physical interventions, such as aerobic exercise, body training, or cognitive training, with a frequency of 1–3 sessions per week, each lasting 30–90 min. The intervention duration ranged from 3 to 12 weeks, depending on the study. Additionally, various technologies were used in the studies, such as wearable devices, mobile phones, and virtual reality devices, providing multiple monitoring and intervention methods for improving the health of older adults. These differences in intervention designs reflect various approaches to improving health outcomes in older adults through wearable technologies and structured physical activity programs.

**Table 2:** Intervention protocol for the dual-arm controlled trial.

Study	Groups	Intervention measures and control details					
		Electronic devices	Intervention	Time	Frequency	Intensity	Cycle
Wu and Manga (2025) (44)	EG	Garmin Vivosmart HR; Apple Watch	Aerobic activity (Walking)	30 min	5 days/week	Moderate-intensity	12 weeks
	CG	Garmin Vivosmart HR; Apple Watch	Maintain the original life	N/A	N/A	N/A	12 weeks
Jung et al. (2025) (45)	EG	RFID	5 min of aerobic exercise; Free physical movements	50 min	2 days/week	N/A	6 weeks
	CG	N/A	Cognition training	50 min	2 days/week	N/A	6 weeks
Shin et al. (2024) (46)	EG	Bot Fit	IWE	52 min	3 days/week	Intensity preferred by the subjects was applied	3 weeks
	CG	N/A	IWE	52 min	3 days/week	Intensity preferred by the subjects was applied	3 weeks
Roberts et al. (2019) (47)	EG	Fitbit Zip	structured exercise	75 min	2 days/week	Moderate-intensity	8 weeks
	CG	N/A	structured exercise	75 min	2 days/week	Moderate-intensity	8 weeks
Lai et al. (2019) (48)	EG	Wearable devices	Resistance Exercise	30 min	1-2 days/week	N/A	12 weeks
	CG	N/A	Routine care	N/A	N/A	N/A	12 weeks
Schwenk et al. (2014) (49)	EG	MatLab^®^ 2007a and Psych toolbox V2.54	Weight shifting and virtual obstacle crossing tasks	45 min	2 days/week	N/A	4 weeks
	CG	N/A	Routine care	N/A	N/A	N/A	4 weeks
Liu et al. (2021) (50)	EG	Fitbit Charge 2	Physical Training	45-60 min	1 days/week	N/A	12 weeks
	CG	N/A	Physical Training	45-60 min	1 days/week	N/A	12 weeks
Kannan et al. (2024) (51)	EG	Electronic remote supervision	Exercise games	90 min	3 days/week	N/A	6 weeks
	CG	N/A	Exercise games	90 min	1 days/week	N/A	8 weeks
Lee et al. (2025) (52)	EG	Mobile health	Outdoor exercise	60 min	1 days/week	N/A	4 weeks
	CG	N/A	interview	N/A	N/A	N/A	4 weeks
Yi et al. (2024) (53)	EG	SHe CoFFEE	Prevention exercises	30 min	3 days/week	N/A	8 weeks
	CG	N/A	Routine care	N/A	N/A	N/A	8 weeks
Kwan et al. (2020) (54)	EG	Mobile phone	Electronic customized exercise	10 min	1 days/week	N/A	12 weeks
	CG	N/A	brisk walking	10 min	1 days/week	N/A	12 weeks
Osuka et al. (2022) (55)	EG	Radio-Taiso	Exercise	90 min	1 days/week	N/A	12 weeks
	CG	N/A	N-exercise	N/A	N/A	N/A	12 weeks
Kwan et al. (2021) (56)	EG	VR Electronic equipment	SMCT	30 min	2 days/week	N/A	6 weeks
	CG	N/A	Cognitive training	30 min	2 days/week	N/A	8 weeks

EG, Experimental Group; CG, Control group; RFID, Arduino and radio-frequency identification; IWE, Interval walking exercise; SMCT, simultaneous motor-cognitive training.

Table 3 summarizes the participant characteristics and intervention protocols in the single-arm trials. The table includes intervention information from four studies, which were conducted in different countries such as South Korea, China, and the United States, and all participants were aged 65 or older. The participants health statuses included healthy, frail, and hypertensive individuals. The interventions primarily involved a combination of exercise, resistance training, and aerobic exercise, with intervention periods ranging from 4 to 20 weeks, and frequency from 3 to 5 days per week, with session durations between 30 min and 150 min. The electronic devices used included assistive robots, AI devices, and digital health platforms to support exercise and health management. The results indicate that exercise interventions based on wearable electronic devices can effectively improve older adults individuals’ physical function, exercise capacity, and quality of life.

**Table 3:** Characteristics of participants in single-arm experiments and intervention protocols.

Study	Country	Sample size (n); Age (years)	Sex	Baseline health status	Electronic devices	Intervention	Cycle; Frequency; Time; Intensity	Outcome indicators	Measuring tools	Outcomes presented in the study
Shin et al. (2023) (57)	Korea	21; ≥65	Male; Female	Healthy	EX1	Combined exercise	4 weeks; 3 days/week; 50 min; N/A	Balance; Muscle strength; WHR	TUG; OLST; Inbody 770	A 4-week exercise program with EX1 was effective in improving the functional gait of the older adults
Seok et al. (2022) (58)	Korea	14; ≥65	Male; Female	Healthy	Context-Aware Artificial Intelligence	Daily exercise	N/A	Physical status; Anthropometrics	SPPB; Horizon DXA System	The TouchCare system is well-accepted by older adults living alone, effectively supporting daily management and health promotion.
Li et al. (2024) (59)	China	16; ≥65	Male; Female	Frail	Digital health platform	Resistance Exercise	8 weeks; 5 days/week; 30 min; N/A	Physical function	30STS; Hand grip Strength test; TUG	Weak older adults people can improve their quality of life on mobile health platforms.
Lefferts et al. (2023) (60)	USA	21; ≥65	Male; Female	hypertension	Electronic supervision	aerobic physical activity	20 weeks; 150 min/week; N/A	exercise motivation	tri-axial accelerometer-based pedometer	E-health can improve the health of the older adults

WHR, Waist-hip ratio; TUG: Including: four-square step test (FSST), functional reach test (FRT), and single leg stance (SLS); OLST, One-leg standing test; SPPB, Short Physical Performance Battery; EX1, Assist robot; STS, sit to stand test.

### 3.3 Methodological quality of included studies

**Figure 3:**
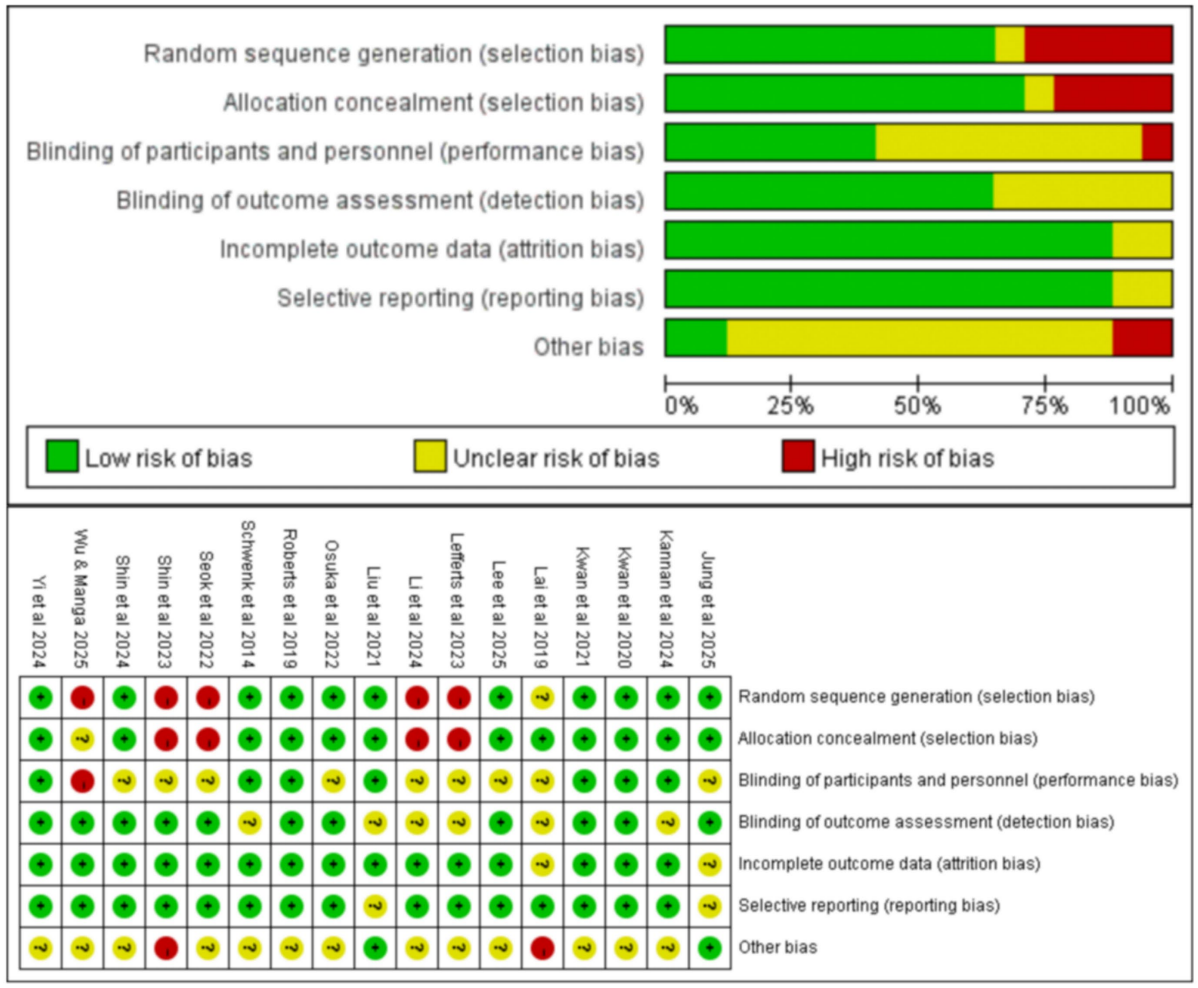
Risk of bias graph and risk of bias summary.

The methodological quality of the included controlled studies was assessed using the Cochrane Risk of Bias tool (RoB 1.0), following the guidance in the Cochrane Handbook (32). Seven domains were evaluated: random sequence generation, allocation concealment, blinding of participants and personnel, blinding of outcome assessment, incomplete outcome data, selective reporting, and other bias. Each domain was judged as low risk, unclear risk, or high risk according to the Cochrane criteria. Overall, most studies were judged to have low risk of bias in blinding of outcome assessment, completeness of outcome data, and selective reporting (Figure 3). Some studies were rated as unclear or high risk in random sequence generation, allocation concealment, and blinding of participants/personnel. For transparency, domain-level judgments for each study are presented in Figure 3. In addition, we defined an overall risk-of-bias classification rule *a priori*: studies were considered overall low risk if all key domains were low risk; overall moderate risk if at least one key domain was unclear risk but none were high risk; and overall high risk if at least one key domain was high risk. This overall classification was used only for descriptive summary purposes, while interpretation focused on domain-level assessments.

### 3.4 Effect of exercise based on wearable electronic devices on lower limb strength and balance in older adults

For older adults, eight two-arm studies evaluated lower-limb strength. Wearable-supported exercise was associated with an improvement in lower-limb strength compared with control (SMD = −0.60; 95% CI [−1.15, −0.05]; *I*^2^ = 81%). Three single-arm pre–post studies reported within-group improvements (SMD = 0.51; 95% CI [0.05, 0.97]; *I*^2^ = 23.5%). As prespecified, single-arm estimates are presented separately and were not combined with controlled effects.

**Figure 4:**
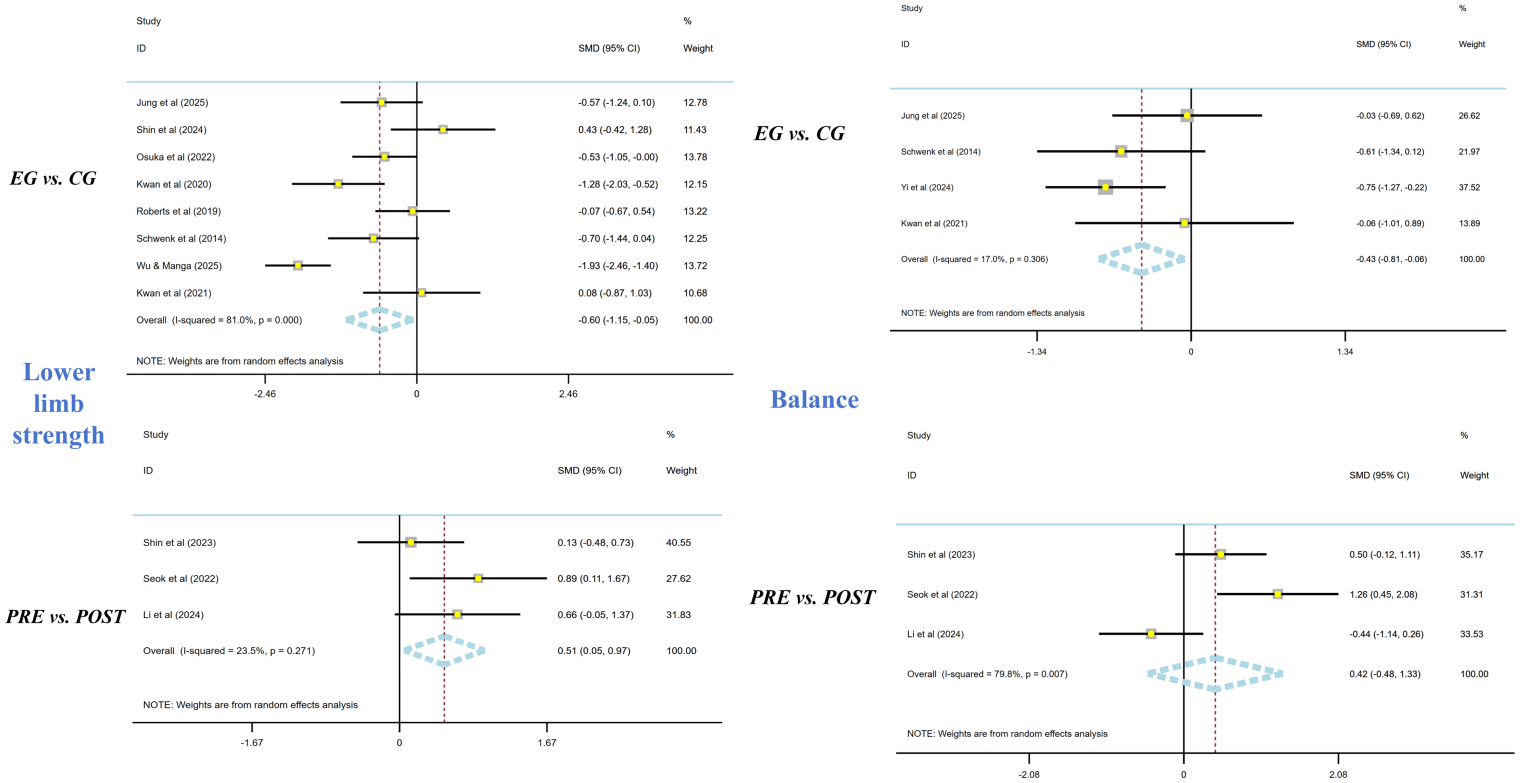
Meta-analysis of the effect of exercise interventions based on wearable electronic devices on lower limb strength and balance in older adults.

Four two-arm studies assessed balance outcomes. A pooled improvement in balance was observed (SMD = −0.43; 95% CI [−0.81, −0.06]; *I*^2^ = 17%). Three single-arm studies reported no statistically significant pre–post change in balance (SMD = 0.42; 95% CI [−0.48, 1.33]; *I*^2^ = 79.8%). Given the small number of studies and substantial heterogeneity, these findings should be interpreted cautiously (Figure 4).

Subgroup analyses were originally conducted to explore heterogeneity for lower-limb strength; however, several subgroups contained very few studies (often *n* = 2). In line with best practice, we now present these subgroup results as exploratory, the results showed that the best improvement was observed with one intervention per week, lasting 10–45 min per session, and a total intervention duration of 8–12 weeks. No other subgroup differences were found. The subgroup tables have been retained for hypothesis generation (Table 4). Meta-regression did not identify intervention frequency, session duration, or intervention period as statistically significant moderators (*p* > 0.05), noting the limited power given the number of available studies.

**Table 4:** Subgroup analysis results of the impact on lower limb strength.

Subgroup		*N*	SMD[95%CI]	*I* ^2^	*P*(subgroup)
Frequency	1 days/week	2	−0.85[−1.58, −0.12]	61.1%	0.109
	2 days/week	4	−0.34[−0.69, 0.02]	0.0%	0.406
	3–5 days/week	2	−0.79[−3.08, 1.53]	95.3%	0.000^***^
Time	10–45 min	3	−1.10[−2.19, −0.02]	84.8%	0.001^**^
	45–60 min	3	−0.32[−0.97, 0.33]	55.6%	0.105
	>60 min	2	−0.32[−0.77, 0.12]	20.2%	0.263
Cycle	<6 week	2	−0.16[−1.27, 0.95]	74.1%	0.049^*^
	6–8 week	3	−0.23[−0.63, 0.18]	0.0%	0.431
	8–12 week	3	−1.24[−2.14, −0.35]	85.3%	0.001^**^

*p < 0.05, **p < 0.01, ***p < 0.001 (test for subgroup differences).

**Figure 5:**
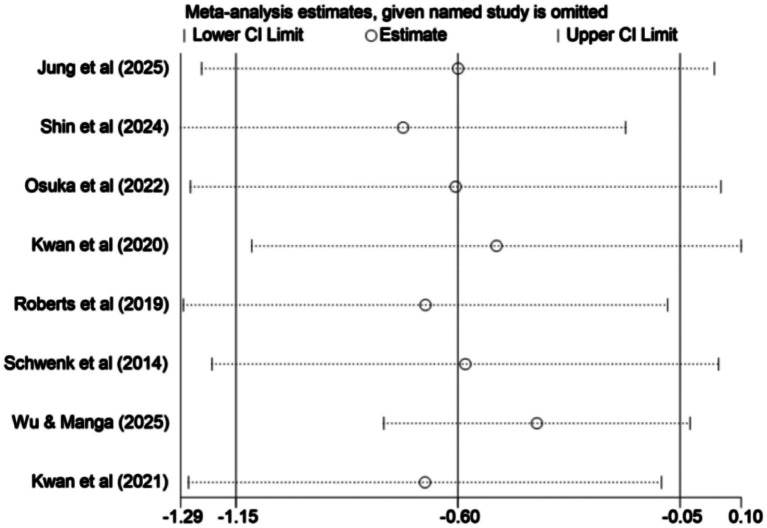
Sensitivity analysis.

**Figure 6:**
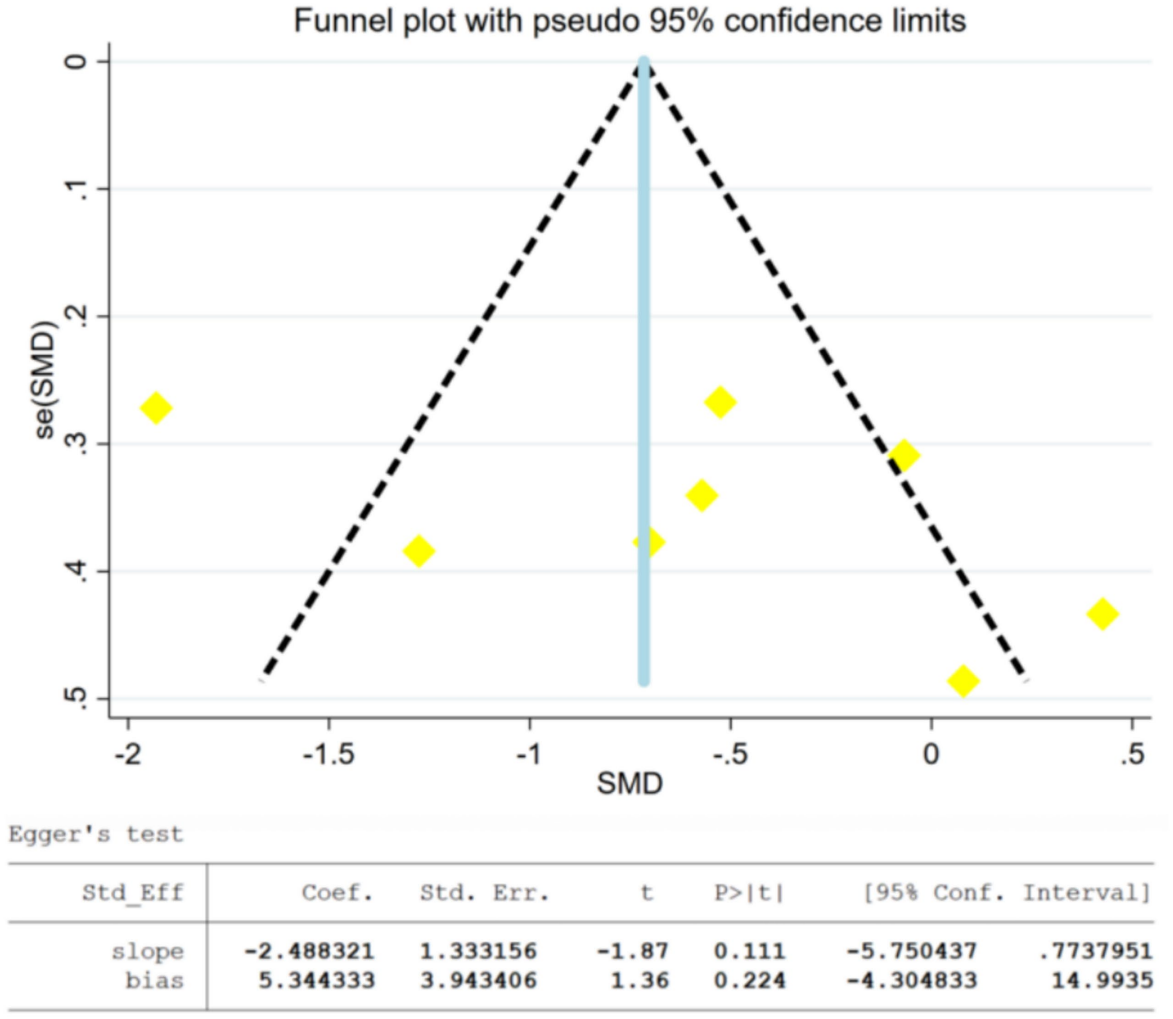
Publication bias funnel plots of included studies and results of Egger’s linear regression method.

To explore the sources of heterogeneity, we conducted a sensitivity analysis (Figure 5), and found that excluding individual studies had minimal impact on the overall effect estimate, indicating high consistency and reliability of this meta-analysis. A funnel plot (Figure 6) was used to assess publication bias. The funnel plot appeared nearly symmetrical; however, given that some outcomes involved fewer than 10 studies, the interpretation of the plot should be considered with caution, as small sample sizes can influence its symmetry. Additionally, the Egger test showed a *p*-value of 0.224 (*p* > 0.05), suggesting that there was no significant publication bias in the study, although this result should be interpreted with caution due to the limited number of included studies. To further examine potential sources of heterogeneity, we performed a meta-regression analysis, which revealed that intervention frequency (*p* = 0.640), duration (*p* = 0.387), and period (*p* = 0.143) were not significant sources of heterogeneity (*p* > 0.05). This strengthens the robustness of the findings and indicates that the observed effects are not influenced by these factors.

### 3.5 Effect of exercise interventions based on wearable electronic devices on endurance, cognitive function, upper limb strength, mental health, and waist-to-hip ratio in older adults

**Figure 7:**
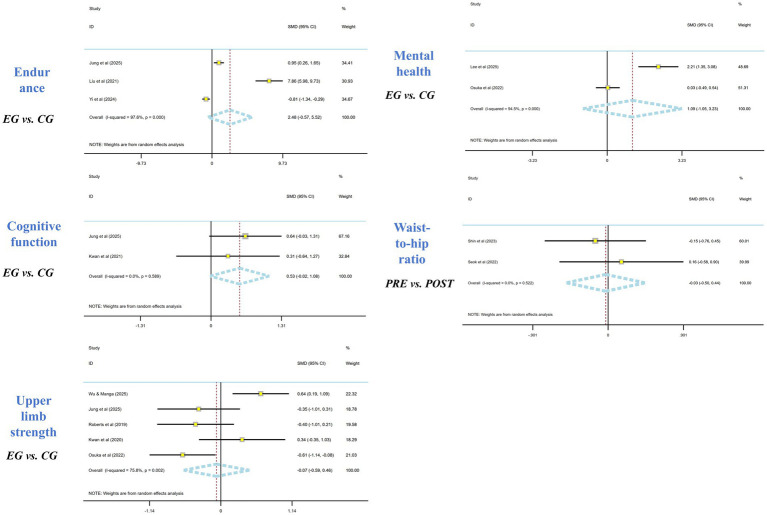
Meta-analysis of exercise interventions based on wearable electronic devices on cardiopulmonary endurance, cognitive function, upper limb strength, mental health, and waist-to-hip ratio in the older adults.

Three two-arm controlled studies reported endurance outcomes. The pooled estimate was not statistically significant and was highly heterogeneous (SMD = 2.48; 95% CI [−0.57, 5.52]; *I*^2^ = 97.6%). Two two-arm controlled studies reported cognitive outcomes. The pooled effect was not statistically significant (SMD = 0.53; 95% CI − 0.02 to 1.08; *I*^2^ = 0.0%). Five two-arm controlled studies reported upper-limb strength outcomes. The pooled effect was not statistically significant (SMD = −0.07; 95% CI −0.59 to 0.46), with high heterogeneity (*I*^2^ = 75.8%). Two two-arm controlled studies reported mental health outcomes. The pooled effect was not statistically significant (SMD = 1.09; 95% CI −1.05 to 3.23), with very high heterogeneity (*I*^2^ = 94.5%). Two single-arm pre–post studies reported waist-to-hip ratio outcomes. The pooled within-group estimate was not statistically significant (SMD = −0.03; 95% CI − 0.50 to 0.90; *I*^2^ = 0.0%). For outcomes with very high heterogeneity (e.g., endurance and mental health), pooled estimates were interpreted cautiously as they reflect substantial between-study variability and imprecision due to the small number of studies (Figure 7).

## 4 Discussion

This systematic review and meta-analysis synthesized evidence on wearable-supported exercise interventions in older adults and found small-to-moderate improvements in lower-limb strength and balance in two-arm controlled trials, while effects on endurance, upper-limb strength, cognitive function, mental health, and waist-to-hip ratio were not statistically significant and were often accompanied by very high heterogeneity and/or a limited number of studies. Taken together, the evidence suggests that wearable-supported exercise may be most consistently associated with improvements in functional outcomes closely linked to mobility and fall-related capability, whereas evidence for broader physiological or psychosocial outcomes remains inconclusive under current study designs and sample sizes.

The observed improvements in lower-limb strength and balance are clinically meaningful because these domains are central determinants of mobility and independence in later life. Importantly, the controlled evidence indicated benefit despite substantial heterogeneity for strength, suggesting that intervention effects may vary across populations (healthy vs. frail), device modalities, and program characteristics. Rather than implying a uniform effect, our findings support a cautious interpretation: wearable-supported exercise interventions can improve strength and balance in some contexts, but the magnitude of benefit is likely contingent on how interventions are delivered and measured (33, 34). These results align with prior research showing that exercise programs emphasizing resistance and balance components can improve functional performance in older adults (23). Our contribution is to summarize the subset of interventions that incorporate wearable monitoring/feedback and quantify their pooled effects on these domains. Nevertheless, the application of exercise combined with wearable electronic devices in the older adult population is still gradually increasing (20, 35, 36).

For endurance, upper-limb strength, cognitive function, mental health, and waist-to-hip ratio, pooled effects were not statistically significant. However, these null findings should be interpreted in the context of limited statistical power and high between-study variability. Several outcomes were informed by only two to five controlled trials, and some showed extreme heterogeneity, indicating that studies likely differed in intervention content (exercise modality, supervision, co-interventions), measurement instruments, and participant health status. Under such conditions, a non-significant pooled estimate does not necessarily indicate absence of effect; it may reflect imprecision and inconsistency in the evidence base. Accordingly, our conclusions for these outcomes are conservative, current evidence is insufficient to establish clear benefits of wearable-supported exercise interventions beyond lower-limb strength and balance. Although wearable devices can theoretically enable individualized feedback and adaptive goal setting, the included studies did not formally test personalization mechanisms, such as algorithm-driven tailoring compared with non-tailored wearable feedback. Therefore, our findings should not be interpreted as evidence that “personalization” or “precision health” components caused the observed benefits. Instead, the current evidence supports a more limited inference, wearable-supported interventions, as implemented in the included trials, are associated with improved lower-limb strength and balance. Future trials should explicitly evaluate whether personalization features (e.g., adaptive intensity targets, individualized feedback rules, or AI-driven coaching) provide incremental benefit over standard wearable monitoring.

Prior reviews of exercise in older adults generally report improvements in functional performance and, in some contexts, cognitive or psychosocial outcomes (25, 37). Our quantitative findings refine this broader literature by indicating that, within the subset of studies incorporating wearable support, evidence is strongest for functional outcomes (lower-limb strength and balance), whereas evidence for endurance, cognitive function, and mental health is currently too limited and heterogeneous for firm conclusions. This distinction is important for clinical and public health translation, if the primary goal is to improve mobility-related function, wearable-supported exercise appears promising, if the goal is cardiometabolic or psychosocial improvement, current wearable-supported evidence does not yet provide consistent support and should be considered exploratory. Wearable electronic devices as health intervention tools, have demonstrated significant potential in managing the health of older adults (38, 39). Through real-time health data monitoring and feedback, older adults can adjust their exercise plans based on their health status, improving exercise adherence and initiative. However, at present, these devices still face several challenges, such as comfort, ease of use, and older adults acceptance of technology (40). Despite ongoing technological advancements, issues such as operational complexity, discomfort in wearing, and battery life may limit the sustained use of these devices (41, 42). Moreover, existing intervention programs are often standardized and lack personalized design, which limits their applicability to different older adults populations (43).

### 4.1 Limitations and future research

Future research should prioritize well-designed controlled trials with adequate sample sizes and standardized outcome assessments for strength and balance, and should also expand high-quality evidence for other domains. To reduce heterogeneity and improve interpretability, trials should report intervention components in detail (exercise modality, supervision, device feedback functions, behavioral strategies) and predefine primary outcomes. Mechanistic questions—particularly whether personalization features add benefit—should be addressed using factorial or head-to-head designs that compare tailored vs. non-tailored wearable support. Finally, longer follow-up and consistent reporting of adverse events will be essential to evaluate sustainability and safety in older populations.

## 5 Conclusion

This systematic review and meta-analysis suggests that wearable-supported exercise interventions are associated with improvements in lower-limb strength and balance in older adults. For other outcomes (e.g., endurance, upper-limb strength, cognitive function, mental health, and waist-to-hip ratio), the current evidence remains inconclusive, largely due to the limited number of studies and substantial heterogeneity in several analyses. Accordingly, while wearable-supported exercise may be a promising approach to enhance mobility-related function, broad claims of overall health benefits or recommendations for widespread implementation are not yet warranted. Future well-designed, adequately powered controlled trials with longer follow-up, standardized outcome assessments, and detailed reporting of device functions and intervention components are needed to clarify effectiveness across domains and to inform practical implementation.

## Data Availability

The anonymized dataset used for analysis will be made available from the corresponding author upon reasonable request.

## Glossary

RCTs: randomized controlled trials
QE: Quasi-experimental
EG: Experimental Group
CG: Control group
SFT: Senior Fitness Test
RCT: randomized clinical trial
MoCA: Montreal cognitive assessment
TUG Including: four-square step test (FSST), functional reach test (FRT), and single leg stance (SLS)
ACT: arm curl test
GS: grip strength
STS: sit to stand test
MWT: minute walk test
MRAK-10: Digital muscle strength measuring instrument
SFT: senior fitness test
SWEMWBS: Short Warwick-Edinburgh Mental Well-Being Scale
SPPB: Short physical performance battery
MCS: mental component summary
MMSE: cognitive status
RFID: Arduino and radio-frequency identification
IWE: Interval walking exercise
SMCT: simultaneous motor-cognitive training
OLST: One-leg standing test
WHR: Waist-hip ratio
